# Supplementary material for: Complexome profiling of the Chlamydomonas psb28 mutant reveals TEF5 as an early PSII assembly factor
Source: Plant Cell. 2025 Mar 17;37(6):koaf055. doi: 10.1093/plcell/koaf055 (PMC12164590; doi:10.1093/plcell/koaf055)
Supplement: koaf055_Supplementary_Data [file koaf055_supplementary_data.zip › psb28_interactive.zip!/plots/intensity/qualityPlots/peptides/ELIP1_Cre16.g679250.t1.2.html]

### Cre16.g679250.t1.2

Trivial name: ELIP1  
SeqCoverage [median]: 10.20  
Mass: 18.34 kDa  
  
 

| Sequence | MissedCleavage | Mass [Da] | wtIntensity | muIntensity | Score |
| --- | --- | --- | --- | --- | --- |
| MEAFGMFTPR | 0 | 1185.53 | 0.00 | 1096.78 | 66.69 |
| VSLFDAMK | 0 | 909.46 | 0.00 | 948.14 | 75.74 |
|  |  |  | 0.0 | 2044.9 |  |
